# Potential value and research frontiers of viruses in inflammatory bowel diseases: a bibliometric analysis

**DOI:** 10.3389/fmicb.2025.1624508

**Published:** 2025-10-15

**Authors:** Wei Zhang, Menglong Zou, Yao Zhou, Qiwei Tang, Ying Zhu, Yin Xu

**Affiliations:** ^1^Department of Gastroenterology, The First Hospital of Hunan University of Chinese Medicine, Changsha, Hunan, China; ^2^Liuyang Hospital of Traditional Chinese Medicine, Changsha, Hunan, China; ^3^Medical School, Hunan University of Chinese Medicine, Changsha, Hunan, China

**Keywords:** inflammatory bowel diseases, Crohn disease, ulcerative colitis, virus, gut virome, opportunistic infections, COVID-19, bibliometric analysis

## Abstract

**Background:**

The pathogenesis of inflammatory bowel diseases (IBD) and their interaction with viruses have attracted growing scientific attention, as viral infections and gut virome dysregulation are increasingly recognized as key drivers of IBD onset, disease activity, and treatment responses. However, a systematic analysis of research trends in this field is lacking, leaving critical gaps in understanding how viral factors shape IBD pathogenesis and clinical management. This study conducted a bibliometric analysis to map trends, key areas, and emerging topics in the virus-IBD field from 2014 to 2024, with a focus on the pathogenic roles of viruses. The goal was to inform future research directions and bridge the gap between basic science and clinical practice.

**Methods:**

Publications related to virus-IBD were retrieved from the Web of Science Core Collection. The analysis employed VOSviewer, CiteSpace, and HistCite software to explore bibliometric dimensions, including annual publication trends, contributions by countries/regions and institutions, collaboration networks, highly cited references, citation bursts, and keyword co-occurrence and evolution.

**Results:**

Among 3,225 publications analyzed, three distinct phases were observed: fluctuating growth (2014–2019), a sharp rise (2020–2021), and a gradual decline (2022–2024). The United States (996 publications) and China (507 publications) were the dominant contributors to the field. The Mayo Clinic led in institutional publication output, while Jean-Frederic Colombel and Silvio Danese were among the most prolific and cited authors. *Inflammatory Bowel Diseases* and *Frontiers in Immunology* were leading journals. Keyword and reference analyses highlighted major research domains: gut virome mechanisms and modulation, clinical strategies for opportunistic viral infections, and IBD management during Coronavirus Disease 2019 (COVID-19).

**Conclusion:**

This study establishes an integrated knowledge framework of virus-IBD research, highlighting three essential domains: gut virome regulation and therapy, clinical management of opportunistic viral infections, and IBD care in the COVID-19 era. It further clarified the pathophysiological interplay between viral factors and IBD. By synthesizing key contributors, core themes, and evolutionary trends, this work provides a practical foundation for guiding translational research and promoting clinical innovation.

## 1 Introduction

Inflammatory Bowel Diseases (IBD), which includes ulcerative colitis (UC) and Crohn disease (CD), is characterized by chronic intestinal inflammation and presents with symptoms such as bloody diarrhea, abdominal pain, and weight loss (Baumgart and Carding, 2007). The global burden of IBD has increased substantially, with an estimated 6.8 million prevalent cases worldwide in 2017 (GBD 2017 Inflammatory Bowel Disease Collaborators, 2020). From 1990 to 2021, the age-standardized incidence rate (ASIR) of IBD increased from 4.22 to 4.45 per 100,000 population, while age-standardized disability-adjusted life years declined from 21.55 to 18.07 per 100,000 population-suggesting improved disease management despite growing incidence. Geographically, East Asia experienced the steepest ASIR increase, particularly in China. Notably, the Netherlands recorded the highest age-standardized mortality rate (2.21 per 100,000) in 2021, underscoring regional disparities in IBD outcomes (Lin et al., 2024).

The pathogenesis of IBD involves the interplay of genetics, immune dysregulation, gut dysbiosis, and environmental factors, though precise mechanisms remain unclear. Genetic factors, particularly Nucleotide-Binding Oligomerization Domain 2 (NOD2) and Autophagy-Related 16-Like 1 (ATG16L1) mutations, are strongly linked to CD (Roda et al., 2020). Immune dysfunction presents as overactive mucosal responses with excessive pro-inflammatory cytokines causing intestinal damage (Friedrich et al., 2019), while gut dysbiosis features reduced beneficial bacteria like *Faecalibacterium prausnitzii* alongside increased pathogens (Cornuault et al., 2018). Environmental triggers include Westernized diets, smoking, and reduced early-life microbial exposure (Piovani et al., 2019). These genetic-environmental interactions disrupt host-microbe crosstalk, triggering IBD onset through intestinal barrier impairment and microbial translocation. Innate immune cells are then activated, releasing cytokines that recruit additional immune cells and initiate adaptive immunity. While regulatory T cells attempt to balance inflammation, chronic activation causes dysregulated cytokine release and immune resistance to anti-inflammatory signals, creating a self-sustaining inflammatory cycle (Neurath, 2019). The COVID-19 pandemic further highlighted critical gaps in IBD management, particularly regarding infection susceptibility, immunotherapy safety, and vaccination strategies in immunosuppressed patients (Ungaro et al., 2021). This global health event reshaped research priorities, intensifying the focus on infection risks and vaccine responses in this population. However, major knowledge gaps persist, limiting therapeutic efficacy and increasing the risks of adverse events (Kobayashi et al., 2020; Roda et al., 2020). Therefore, there is an urgent need for further research into IBD mechanisms and novel therapeutic strategies.

Current management strategies for IBD include 5-aminosalicylates, glucocorticoids, immunomodulators (e.g., azathioprine), and biologic agents targeting pro-inflammatory pathways. Diagnosis relies on clinical assessment, endoscopic evaluation of mucosal lesions, histopathological analysis of biopsies, and serological markers such as antineutrophil cytoplasmic antibody (ANCA) and anti-Saccharomyces cerevisiae antibody (ASCA) (Nakase et al., 2021; Khrom et al., 2024). Recent metagenomic advances underscore the gut microbiota’s influence on metabolism and immune function, with dysbiosis linked to IBD. Fecal microbiota transplantation helps correct this gut dysbiosis (Sartor and Wu, 2017). While bacterial communities are well-studied, the role of the gut virome in IBD pathogenesis is increasingly recognized (Wu et al., 2025). Virome imbalance, characterized by an increase in Caudovirales and a decrease in Microviridae, is associated with intestinal barrier disruption and aberrant immune activation. Certain viruses, such as *Orthohepadnavirus*, may worsen UC by damaging the epithelium (Tun et al., 2024). Various viruses, including bacteriophages, rotaviruses, and noroviruses, are involved in IBD (Tarris et al., 2021). Pediatric studies have revealed distinct virome patterns in CD and UC, indicating a significant role for viruses in the pathogenesis of IBD (Fernandes et al., 2019). These findings highlight the virome as a potential therapeutic target. However, virus-IBD research faces challenges in establishing causal inference, including unconfirmed causal links and the risk of secondary infections from immunosuppressive therapies (Rahier et al., 2014; Reddy et al., 2015).

Despite the growing body of research on viruses and IBD, a comprehensive bibliometric analysis of this field remains lacking. This gap is critical because bibliometric analysis helps identify emerging themes and underexplored topics amid the rapid growth of this field (Chen and Song, 2019). Unlike previous bibliometric analyses that focused broadly on gut microbiota or single viral taxa, our study is the first to perform a decade-long bibliometric analysis specifically targeting virus-IBD associations (Veisman et al., 2022; Zhang et al., 2022b,^2023^). By quantifying collaboration networks, tracking keyword evolution, and analyzing citation patterns, we aim to delineate research paradigms, highlight translational gaps, and inform future directions for both basic research and clinical applications.

## 2 Materials and methods

### 2.1 Data source and search strategy

A systematic literature search was conducted on August 30, 2025, at 20:38 Beijing Time (UTC + 8) using the Web of Science Core Collection (WoSCC) database, specifically the Science Citation Index Expanded (SCI-Expanded). WoSCC was selected for this bibliometric analysis due to its rigorous citation indexing, which ensures data integrity by excluding predatory and low-quality journals; its KeyWords Plus feature, which supplements author keywords and captures an additional 40%–60% of semantic linkages; and its native compatibility with analytical tools such as VOSviewer, which facilitates efficient data normalization.

The literature search encompassed publications from January 1, 2014, to December 31, 2024. This 10-year span was selected because it represents a recent and complete decade, which helps capture evolving research trends without including outdated studies. The timeframe is sufficiently long to identify meaningful patterns yet focused enough to maintain relevance and analytical consistency. The search strategy employed the following Boolean query: #1: “Inflammatory Bowel Disease*” OR IBD OR “Ulcerative Colitis” OR “Crohn* Disease” OR “Crohn’s Disease”; #2: Virus* OR Virology OR Virological OR Viral OR Virome OR Bacteriophage* OR Phage* OR Herpesvirus* OR Enterovirus* OR Retrovirus* OR Norovirus* OR Adenovirus* OR Rotavirus* OR “SARS-CoV-2” OR “COVID-19” OR “Epstein-Barr Virus” OR EBV OR “Cytomegalovirus” OR CMV OR “Human Immunodeficiency Virus” OR HIV OR “Hepatitis B Virus” OR HBV OR “Hepatitis C Virus” OR HCV OR “Varicella-Zoster Virus” OR VZV OR “JC Virus” OR JCV; #1 AND #2.

The search terms were informed by Medical Subject Headings (MeSH) descriptors, including [Inflammatory Bowel Diseases] (MeSH), and were supplemented with free-text variants. The TS (Topic Search) field encompasses titles, abstracts, author keywords, and KeyWords Plus, with wildcard truncation (e.g., “inflammatory bowel disease*”) employed to capture grammatical variants such as singular and plural forms.

This analysis included all retrieved literature relevant to viruses and IBD without preemptively excluding any virus type. Studies related to SARS-CoV-2 were retained even when IBD was not the central focus, as they represent a substantive thematic trend and contribute to the overall knowledge structure of the field, thereby aligning with the objective nature of bibliometric analysis.

### 2.2 Inclusion and exclusion criteria

Inclusion criteria: The study considered publications that met the following criteria: (1) Papers published in the English language; (2) Articles or reviews; (3) Studies examining the relationship between viruses and IBD; (4) Publication date between January 1, 2014, and December 31, 2024.

Exclusion criteria: Publications were excluded if they met any of the following conditions: (1) non-English language publications; (2) Document types other than articles and reviews (e.g., letters, meeting abstracts, and editorial materials); (3) Publications falling outside the specified timeframe; (4) Duplicate records; (5) Retracted publications; (6) Publications with irrelevant or incomplete content.

The collected data, including complete bibliometric records and citation information, were stored in plain text format. A three-tier quality control was implemented as follows: (1) Deduplication: When downloading raw data from WoSCC, Excel files containing “authors, titles, and sources” were saved simultaneously. Duplicates were removed based on author-title combinations using Microsoft Excel; (2) Resolution of data inconsistencies was achieved through verification against source metadata; (3) Misclassifications were addressed by re-evaluating publications against predefined criteria. To ensure accuracy, all publications underwent a dual-reviewer screening process: an initial assessment of titles and abstracts in accordance with the inclusion criteria, followed by a comprehensive evaluation of full texts. Any discrepancies were resolved through consensus with a third reviewer.

### 2.3 Data normalization and disambiguation

#### 2.3.1 Keyword normalization

To ensure the accuracy and consistency of keyword co-occurrence analysis, a standardized workflow was applied to filter, clean, and normalize keywords. The synonym list and high-frequency keywords (both pre- and post-merger) are provided in Supplementary materials. The normalization procedure consisted of the following steps:

Keywords with a frequency of ≥15 were extracted using VOSviewer to focus on core research topics and minimize noise from infrequent terms. Each keyword was individually reviewed against the MeSH thesaurus to develop a synonym library. Synonyms with identical meanings were merged, while near-synonyms were retained as distinct entries to preserve conceptual nuance and avoid artificial conflation.

The keyword set underwent three normalization steps before being re-analyzed in VOSviewer: (1) Semantic unification: Variants with identical or highly similar meanings were mapped to standardized MeSH descriptors (e.g., “Crohn’s disease” → “Crohn disease”; “inflammatory-bowel-disease” → “inflammatory bowel diseases”). (2) Abbreviation standardization: Common medical abbreviations were expanded to full MeSH-preferred terms (e.g., “ibd” → “inflammatory bowel diseases”). Widely recognized abbreviations (e.g., “COVID-19”) were retained. (3) Grammatical and structural consistency: Singular/plural forms were unified based on MeSH conventions or prevailing usage (e.g., “infection” → “infections”). Incomplete terms were expanded into full semantic expressions (e.g., “short-chain fatty acids” from “chain fatty-acids”).

#### 2.3.2 Author/institution disambiguation

Author information was extracted using VOSviewer, with a focus on 238 authors who had at least five publications. To resolve name variants (e.g., abbreviations or inconsistent name order), each author’s identity was cross-verified against Open Researcher and Contributor ID (ORCID) profiles and WoS records. VOSviewer-generated default formats (e.g., “farraye, francis a,” “ng, siew c”) were retained in visualizations, while full academic names (e.g., “Francis A. Farraye,” “Siew C. Ng”) were used in the text and tables based on WoS and ORCID records.

For institutional disambiguation, VOSviewer abbreviations were retained in figures but expanded to official full names elsewhere using the Research Organization Registry (ROR) and institutional websites (e.g., “univ coll cork” → “University College Cork”). Among the 97 institutions with at least 15 publications, no duplicates were identified. Four affiliated institutions–Brigham and Women’s Hospital, Massachusetts General Hospital, Harvard Medical School, and Harvard University–were treated as distinct entities due to their operational independence and distinct research profiles, thereby avoiding conflation of their contributions.

### 2.4 Bibliometric analysis

This study utilized a comprehensive array of domain-specific bibliometric tools for a multi-layered analysis. The tools and their functions included: (1) VOSviewer (version 1.6.20) for analyzing collaboration networks and keyword mapping, with Pajek for topology optimization; (2) CiteSpace (version 6.4.R1) for co-citation and the detection of emerging trends via Kleinberg’s burst detection algorithm; (3) HistCite Pro (version 2.1) for evaluating citation impact through both Local Citation Score (LCS), which measures citations received from within the curated dataset of 3,225 publications, and Global Citation Score (GCS), which reflects total citations from the entire WoSCC database. This distinction allows for separate interpretation of a publication’s influence within the specific research domain versus its broader academic impact; (4) Microsoft Excel 2016 for data preprocessing.

**FIGURE 1:**
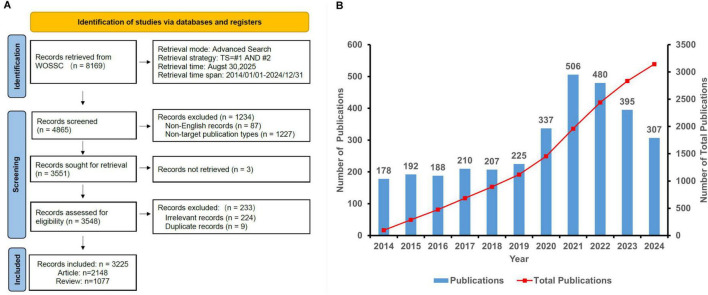
**(A)** Flow chart illustrating the literature screening process. **(B)** Annual publication trends in virus-IBD research (2014–2024).

Moreover, for the analysis of productivity by country, institution, and author, we employed full counting methodology. This means that each publication was counted in full for every contributing country, institution, or author. Figure 1A illustrates this literature screening workflow, highlighting critical decision points from initial identification to final inclusion.

## 3 Results

### 3.1 Analysis of publications

This study analyzed 3,225 publications (2,127 articles and 1,068 reviews) in the virus-IBD field. Figure 1B illustrates a three-phase publication trend from 2014 to 2024: fluctuating growth (2014–2019), a sharp surge (2020–2021), and a gradual decline (2022–2024).

### 3.2 Analysis of countries/regions

A total of 99 countries/regions contributed to the virus-IBD research field. The top 20 most productive countries are presented in Table 1, ranked by publication count. The United States of America (USA) led with 996 publications, accounting for 30.88% of the total publications, followed by China (507 publications, 15.72%), Italy (338 publications, 10.48%), the United Kingdom (UK, 305 publications, 9.46%), and Germany (211 publications, 6.54%). A positive correlation was observed between national publication output and total GCS, though considerable disparities existed in average GCS. The USA maintained a high average GCS (57.98), while Canada (58.29) and Australia (56.98) achieved comparable or even higher average GCS despite lower total publication output. In contrast, China (27.50) and South Korea (26.05) had relatively lower average GCS despite high productivity. In terms of LCS, average LCS varied more notably: Belgium led with 5.67, followed by France (4.99), Canada (4.88), and Portugal (4.50). Conversely, Iran (0.32), Poland (1.05), India (1.27), and Japan (1.20) showed the lowest average LCS, indicating regional differences in scholarly influence.

**TABLE 1:** The top 20 productive countries by publications in virus-IBD research.

Rank	Country	Publications	Percentage	LCS	Average LCS	GCS	Average GCS
1	USA	996	30.88%	3375	3.39	57748	57.98
2	China	507	15.72%	951	1.88	13944	27.50
3	Italy	338	10.48%	912	2.70	10727	31.74
4	United Kingdom	305	9.46%	1309	4.29	14755	48.38
5	Germany	211	6.54%	654	3.10	8570	40.62
6	Canada	190	5.89%	927	4.88	11075	58.29
7	France	179	5.55%	893	4.99	9160	51.17
8	Japan	166	5.15%	199	1.20	5042	30.37
9	Spain	147	4.56%	311	2.12	5615	38.20
10	Australia	120	3.72%	293	2.44	6838	56.98
11	Netherlands	103	3.19%	242	2.35	4415	42.86
12	India	102	3.16%	130	1.27	3503	34.34
13	Belgium	86	2.67%	488	5.67	3403	39.57
14	South Korea	82	2.54%	263	3.21	2136	26.05
15	Israel	77	2.39%	203	2.64	2567	33.34
16	Poland	76	2.36%	80	1.05	1885	24.80
17	Switzerland	68	2.11%	177	2.60	3372	49.59
18	Denmark	60	1.86%	124	2.07	1637	27.28
19	Iran	60	1.86%	19	0.32	1801	30.02
20	Portugal	58	1.80%	261	4.50	2650	45.69

**FIGURE 2:**
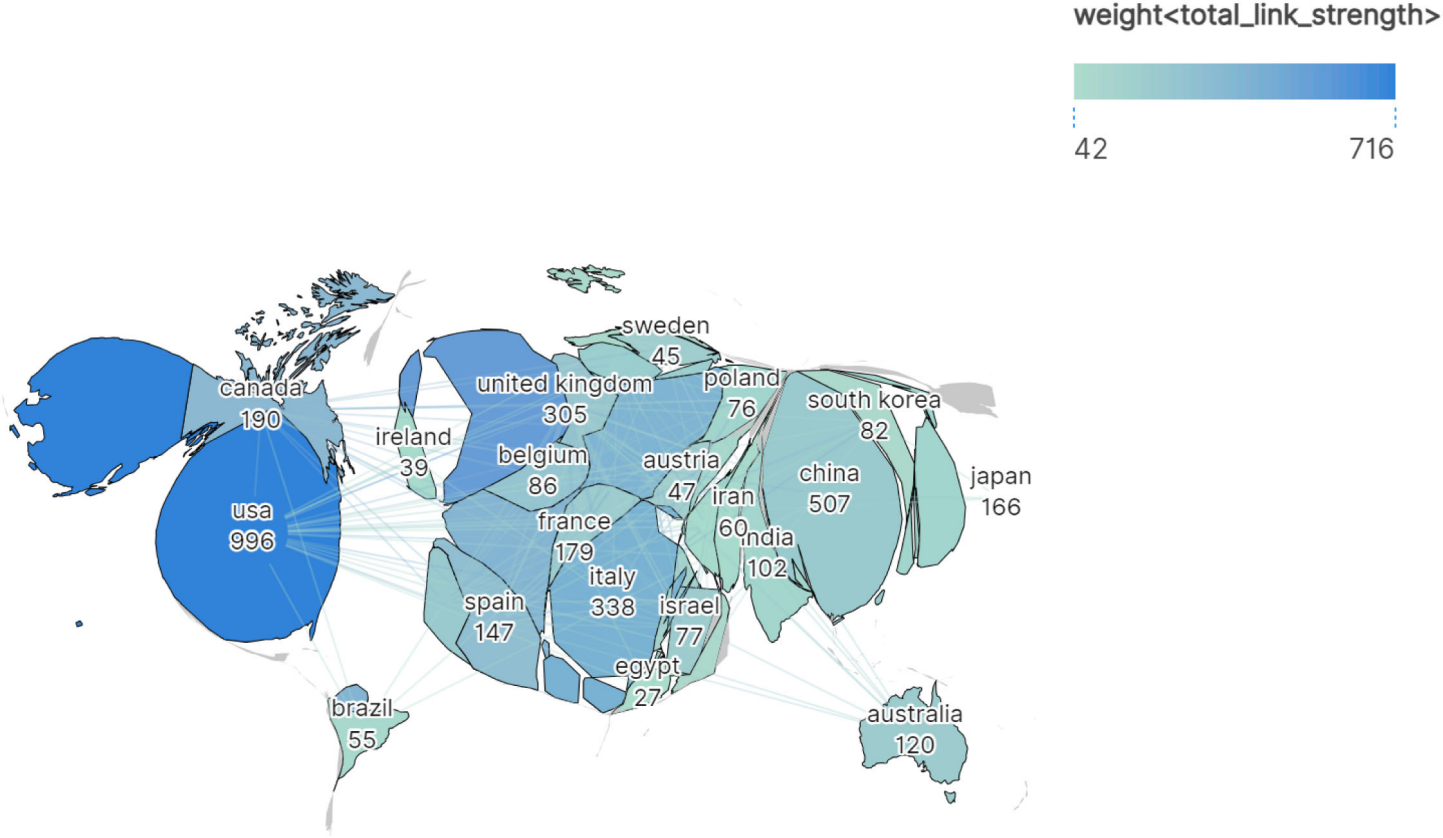
Geospatial collaboration map of publication output and international research networks in the virus-IBD field. Color intensity indicates link strength; geographic area is proportional to publication volume.

Figure 2 presents a geospatial map depicting a part of the top 30 countries ranked by publication output (≥27 publications each), where country size was scaled according to publication volume. The visualization highlights strong collaborative networks among developed regions–particularly the USA, Canada, and major European nations–which dominated both productivity and collaboration. The East Asian countries (e.g., China, Japan, South Korea) also contributed substantially, alongside emerging economies such as India, Iran, and Brazil.

### 3.3 Contribution of institutions

A total of 4,749 institutions contributed to this field. As listed in Table 2, the top 20 most productive institutions (each with ≥28 publications) were identified. Institutions from the USA predominated (13 out of 20), followed by China and Canada (3 each), Israel (1), Italy (2), and the UK (1).

**TABLE 2:** The top 20 institutes by publications in virus-IBD research.

Rank	Institute	Country	Publications	LCS	Average LCS	GCS	Average GCS
1	Mayo Clinic	USA	73	539	7.38	2447	33.52
2	Icahn School of Medicine at Mount Sinai	USA	64	723	11.30	3223	50.36
3	Harvard Medical School	USA	60	116	1.93	1910	31.83
4	University of Pennsylvania	USA	58	591	10.19	3740	64.48
5	University of Toronto	Canada	43	115	2.67	1921	44.67
6	Tel Aviv University	Israel	41	115	2.80	1330	32.44
7	University of California, San Francisco	USA	40	128	3.20	9624	240.60
8	The Chinese University of Hong Kong	China	36	292	8.11	3350	93.06
9	McGill University	Canada	35	495	14.14	1331	38.03
10	University of Milan	Italy	34	95	2.79	661	19.44
11	Sun Yat-sen University	China	32	66	2.06	1106	34.56
12	Shanghai Jiao Tong University	China	31	252	8.13	594	19.16
13	University of Calgary	Canada	31	55	1.77	2681	86.48
14	University of Michigan	USA	31	208	6.71	1566	50.52
15	University of Oxford	United Kingdom	31	70	2.26	1039	33.52
16	Humanitas University	Italy	29	486	16.76	758	26.14
17	University of Washington	USA	29	547	18.86	1879	64.79
18	University of Wisconsin System	USA	29	80	2.76	1206	41.59
19	The Ohio State University	USA	28	437	15.61	1894	67.64
20	University of North Carolina	USA	28	13	0.46	3265	116.61

Key observations include: (1) Publication output: The Mayo Clinic led with 73 publications, followed by the Icahn School of Medicine at Mount Sinai (64) and Harvard Medical School (60); (2) LCS: In terms of total LCS, the Icahn School of Medicine at Mount Sinai ranked highest with 723, followed by the University of Pennsylvania (591) and the University of Washington (547). For average LCS, the University of Washington led with 18.86, followed by Humanitas University (Italy, 16.76) and The Ohio State University (15.61); (3) GCS: In terms of total GCS, the University of California, San Francisco (UCSF) ranked first with 9,624, followed by the University of Pennsylvania (3,740) and The Chinese University of Hong Kong (3,350). For average GCS, UCSF still took the lead with 240.60, followed by the University of North Carolina (116.61) and The Chinese University of Hong Kong (93.06).

Several institutions-including UCSF, the University of North Carolina, and the Chinese University of Hong Kong–demonstrated high average citation metrics, reflecting strong scholarly impact relative to output.

**FIGURE 3:**
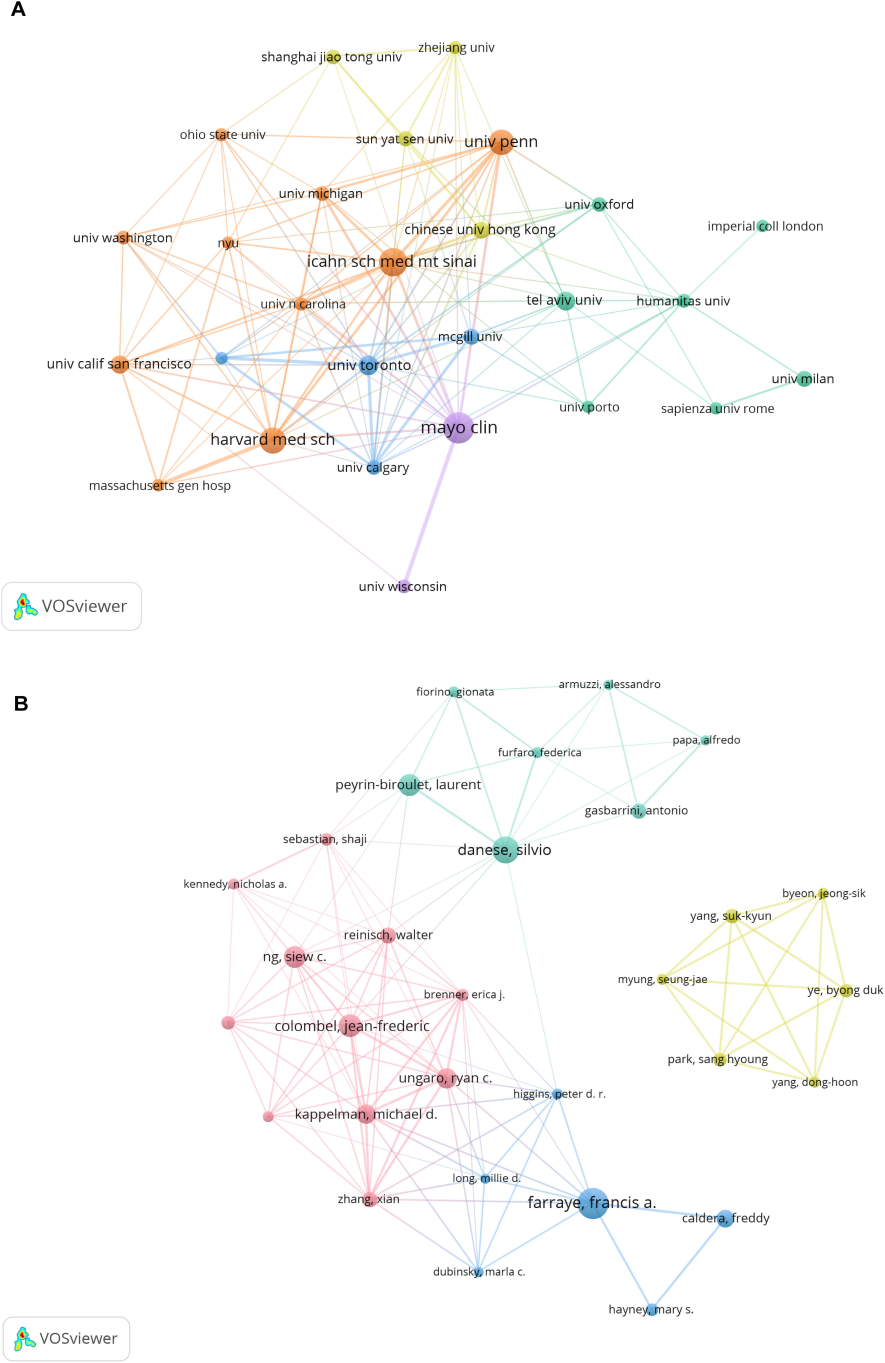
**(A)** Institutional and **(B)** author collaborative networks in virus-IBD research. Node size corresponds to publication count; line width denotes collaborative strength. The analysis included institutions with ≥28 publications **(A)** and authors with ≥10 publications **(B)**. Normalization: association strength. Layout settings: **(A)** attraction = 3, repulsion = –3; **(B)** attraction = 2, repulsion = –1.

Figure 3A displays the collaborative network among institutions. Major hubs-including the Mayo Clinic, Icahn School of Medicine, Harvard Medical School, and the University of Pennsylvania-show extensive collaborations, as indicated by large node size and dense linkage patterns.

### 3.4 Analysis of authors

A total of 18,255 authors contributed to the 3,225 publications. Table 3 lists the top 20 most prolific authors (each with ≥12 publications). Francis A. Farraye (USA) led with 37 publications, followed by Silvio Danese (Italy) with 31, and Jean-Frederic Colombel (USA) with 26. In terms of total LCS, Shaji Sebastian (UK) ranked first with 645 points and also had the highest average LCS (49.62), followed by Michael D. Kappelman (USA, with a total LCS of 518 and an average LCS of 24.67) and Ryan C. Ungaro (USA, with a total LCS of 493 and an average LCS of 22.41). Regarding GCS, Siew C. Ng (China) took the lead with a total GCS of 2704, followed by Jean-Frederic Colombel (USA, with a total GCS of 1926 and an average GCS of 74.08) and Gilaad G. Kaplan (Canada, with a total GCS of 1852). For average GCS, Gilaad G. Kaplan topped the list with 132.29, followed by Siew C. Ng (112.67) and Erica J. Brenner (USA, 77.58) and Colombel (74.08). The H-index, a metric reflecting sustained scholarly influence, was highest for Jean-Frederic Colombel (135), followed by Silvio Danese (120) and Antonio Gasbarrini (103).

**TABLE 3:** The top 20 authors in virus-IBD research.

Rank	Author	Country	Institute	Publications	LCS	Average LCS	GCS	Average GCS	H-index
1	Francis A. Farraye	USA	Mayo Clinic	37	300	8.11	945	25.54	54
2	Silvio Danese	Italy	Vita-Salute San Raffaele University	31	289	9.32	1021	32.94	120
3	Jean-Frederic Colombel	USA	Icahn School of Medicine at Mount Sinai	26	458	17.62	1926	74.08	135
4	Siew C. Ng	China	The Chinese University of Hong Kong	24	488	20.33	2704	112.67	85
5	Laurent Peyrin-Biroulet	France	University of Nancy	24	214	8.92	655	27.29	92
6	Ryan C. Ungaro	USA	Icahn School of Medicine at Mount Sinai	22	493	22.41	1106	50.27	43
7	Michael D. Kappelman	USA	University of North Carolina at Chapel Hill	21	518	24.67	1172	55.81	60
8	Freddy Caldera	USA	Medical College of Wisconsin	19	101	5.32	220	11.58	19
9	Walter Reinisch	Austria	Medical University of Vienna	17	371	21.82	1020	60.00	90
10	Antonio Gasbarrini	Italy	Università Cattolica del Sacro Cuore	16	47	2.94	388	24.25	103
11	Xian Zhang	USA	University of North Carolina Chapel Hill	16	453	28.31	1033	64.56	15
12	Suk-Kyun Yang	South Korea	University of Ulsan	15	115	7.67	366	24.40	53
13	Gilaad G. Kaplan	Canada	University of Calgary	14	398	28.43	1852	132.29	77
14	Sang Hyoung Park	South Korea	University of Ulsan	14	95	6.79	278	19.86	74
15	David T. Rubin	USA	The University of Chicago Medical Center	14	221	15.79	648	46.29	68
16	Byong Duk Ye	South Korea	Asan Medical Center, University of Ulsan	14	115	8.21	296	21.14	46
17	Mary S. Hayney	USA	University of Wisconsin Madison	13	85	6.54	178	13.69	24
18	Shaji Sebastian	UK	Hull University Teaching Hospitals NHS Trust	13	645	49.62	618	47.54	47
19	Hong Yang	China	Chinese Academy of Medical Sciences	13	38	2.92	151	11.62	20
20	Erica J. Brenner	USA	University of North Carolina Chapel Hill	12	376	31.33	931	77.58	14

Figure 3B illustrates the co-authorship network among the top 30 authors, revealing four collaborative clusters. The cluster represented by Jean-Frederic Colombel, Silvio Danese, and Francis A. Farraye exhibits strong internal collaborations, whereas the cluster centered around Suk-Kyun Yang (South Korea) was relatively isolated, showing limited connections to researchers from other clusters.

### 3.5 Analysis of journals

A total of 966 journals were represented in the virus-IBD research. Table 4 presents the top 20 journals by publication volume. *Inflammatory Bowel Diseases* led with 135 articles, followed by *Frontiers in Immunology* (100) and *Journal of Crohn’s & Colitis* (81). In terms of GCS, Gastroenterology received the highest total GCS (6,116), far exceeding other journals, and achieved the highest average GCS (244.64). *Frontiers in Immunology* ranked second in total GCS (3,931), while Gut recorded the second-highest average GCS (113.15). Regarding *journal influence metrics, Gut and Gastroenterology* led with the highest Impact Factors (25.8 and 25.1, respectively). Among the H5 index values, which reflect recent scholarly impact, *International Journal of Molecular Sciences scored highest* (277), followed by *PLOS One* (244) and *Scientific Reports* (234). These metrics collectively underscore the significant role of these *journals in disseminating high-impact virus-IBD research*.

**TABLE 4:** The top 20 leading journals in virus-IBD research.

Rank	Journal	Total articles	GCS	Average GCS	IF (2024)	H5 index
1	*Inflammatory Bowel Diseases*	135	3690	27.33	4.3	61
2	*Frontiers in Immunology*	100	3931	39.31	5.9	224
3	*Journal of Crohn’s* & *Colitis*	81	2377	29.35	8.7	78
4	*World Journal of Gastroenterology*	70	2707	38.67	5.4	84
5	*Digestive Diseases and Sciences*	48	869	18.10	2.5	58
6	*Journal of Clinical Medicine*	41	424	10.34	2.9	159
7	*International Journal of Molecular Sciences*	39	1090	27.95	4.9	277
8	*Alimentary Pharmacology* & *Therapeutics*	37	1489	40.24	6.7	77
9	*Scientific Reports*	36	414	11.50	3.9	234
10	*European Journal of Gastroenterology* & *Hepatology*	34	375	11.03	1.8	37
11	*Vaccines*	34	211	6.21	3.4	122
12	*Frontiers in Medicine*	32	312	9.75	3.0	114
13	*Frontiers in Microbiology*	31	1070	34.52	4.5	156
14	*PLOS One*	28	597	21.32	2.6	244
15	*Journal of Pediatric Gastroenterology and Nutrition*	27	544	20.15	2.6	44
16	*American Journal of Gastroenterology*	26	1416	54.46	7.6	85
17	*Gut*	26	2942	113.15	25.8	167
18	*Viruses-Basel*	26	603	23.19	3.5	113
19	*Gastroenterology*	25	6116	244.64	25.1	176
20	*BMC Gastroenterology*	24	227	9.46	2.6	50

**FIGURE 4:**
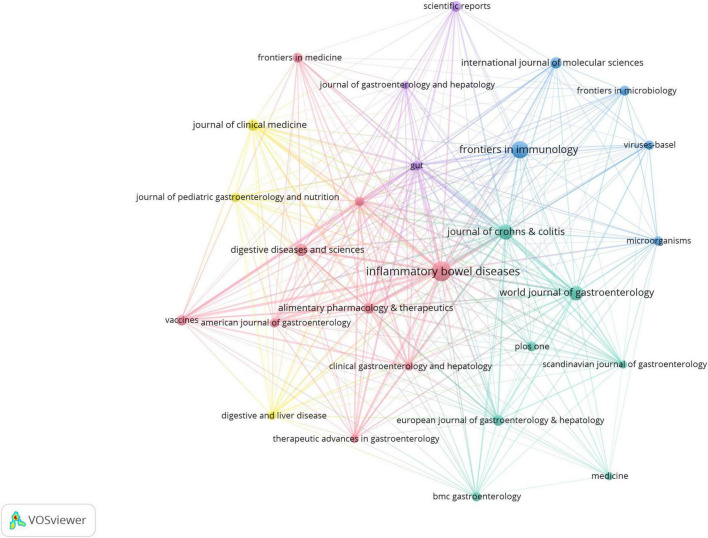
Journal citation network in virus-IBD research. Node size corresponds to the publication output; line width indicates citation strength between journals. Journals with ≥20 publications were included. Normalization: association strength. Layout settings: attraction = 2; repulsion = –1.

Figure 4 presents a journal citation network based on an analysis of 27 journals (each with ≥20 publications). The visualization highlights several core journals in this field, including *Inflammatory Bowel Diseases*, *Frontiers in Immunology*, *World Journal of Gastroenterology*, and *Journal of Crohn’s* & *Colitis*.

### 3.6 Analysis of highly cited references and burst

To pinpoint core foundational studies in virus-IBD research, we extracted the top 10 LCS using HistCite, detailed in Table 5. These publications reflect several prominent research themes: (1) Clinical management and guidelines for opportunistic infections and COVID-19 in patients with IBD (Rahier et al., 2014; Bezzio et al., 2020; Brenner et al., 2020; Kennedy et al., 2020; Ungaro et al., 2021); (2) Vaccine immunogenicity and outcomes in IBD (Polack et al., 2020; Kennedy et al., 2021); (3) Viral ecology in IBD, like enteric virome changes (Norman et al., 2015); (4) Epidemiology and infection risks associated with IBD treatments (Ng et al., 2017; Kirchgesner et al., 2018). The most cited reference was the second European consensus statement on opportunistic infections in IBD (LCS = 251) (Rahier et al., 2014), followed by a study on COVID-19 outcomes under corticosteroid therapy (LCS = 202) (Brenner et al., 2020), and a study on enteric virome changes (LCS = 158) (Norman et al., 2015). Notably, five of the top ten publications focused on COVID-19, highlighting its significant impact on recent IBD research.

**TABLE 5:** The top 10 cited local publications in virus-IBD research based on LCS.

Rank	First Author	Year	Title	LCS	Journal
1	Jean F. Rahie	2014	Second European evidence-based consensus on the prevention, diagnosis and management of opportunistic infections in inflammatory bowel disease	251	*Journal of Crohns* & *Colitis*
2	Erica J. Brenner	2020	Corticosteroids, but not TNF antagonists, are associated with adverse COVID-19 outcomes in patients with inflammatory bowel diseases: results from an international registry	202	*Gastroenterology*
3	Joshua M. Norman	2015	Disease-specific alterations in the enteric virome in inflammatory bowel disease	158	*Cell*
4	Claudio Bezzio	2020	Outcomes of COVID-19 in 79 patients with IBD in Italy: an IG-IBD study	127	*GUT*
5	Niamh A. Kennedy	2021	Infliximab is associated with attenuated immunogenicity to BNT162b2 and ChAdOx1 nCoV-19 SARS-CoV-2 vaccines in patients with IBD	112	*GUT*
6	Rachael C. Ungaro	2021	Effect of IBD medications on COVID-19 outcomes: results from an international registry	112	*GUT*
7	Siew C. Ng	2017	Worldwide incidence and prevalence of inflammatory bowel disease in the 21st century: a systematic review of population-based studies	109	*Lancet*
8	Julien Kirchgesner	2018	Risk of serious and opportunistic infections associated with treatment of inflammatory bowel diseases	101	*Gastroenterology*
9	Niamh A. Kennedy	2020	British society of gastroenterology guidance for management of inflammatory bowel disease during the COVID-19 pandemic	99	*GUT*
10	Francis P. Polack	2020	Safety and Efficacy of the BNT162b2 mRNA Covid-19 vaccine	99	*New England Journal of Medicine*

**FIGURE 5:**
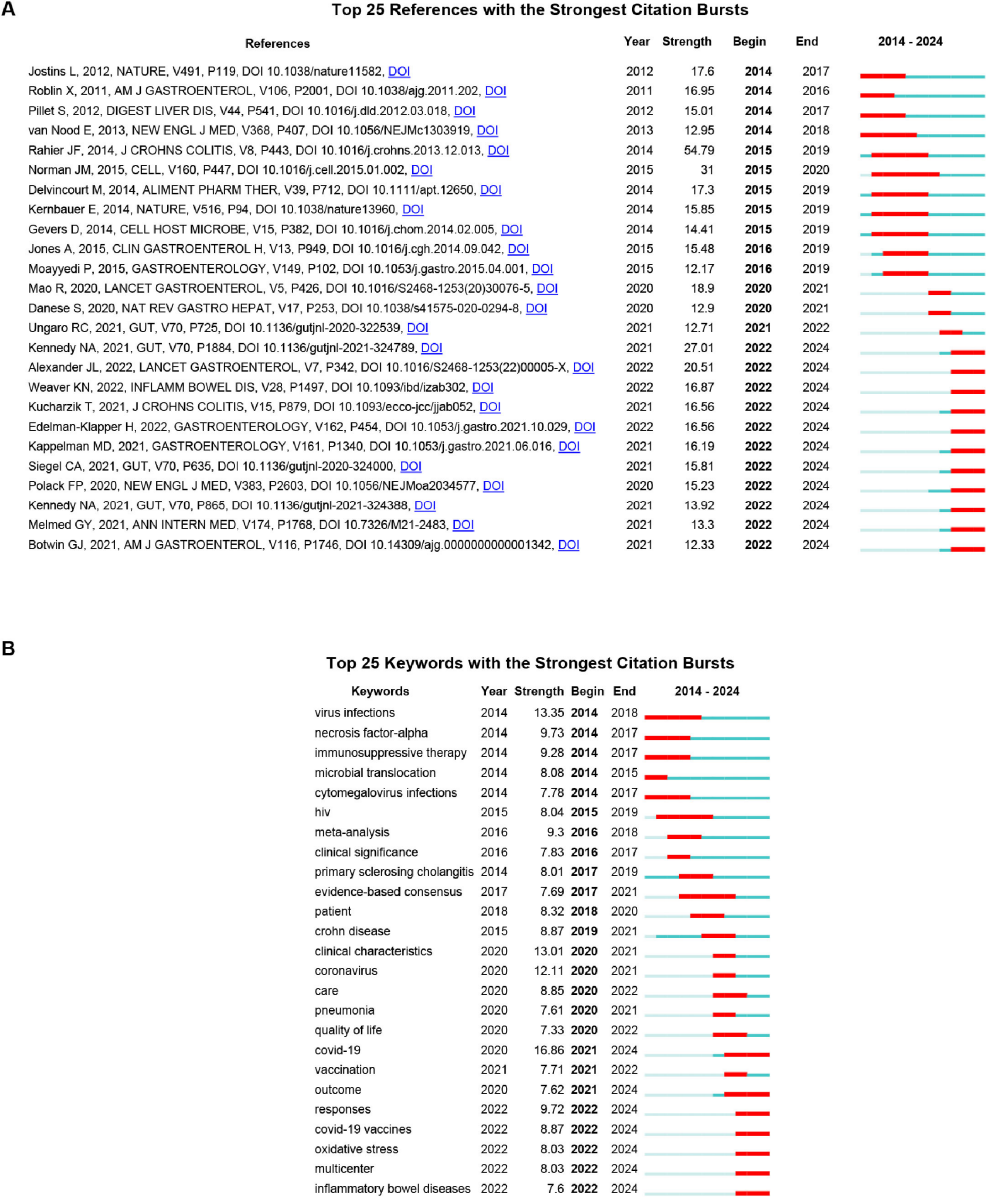
**(A)** Top 25 references with the strongest citation bursts. **(B)** Top 25 keywords with the strongest citation bursts. Blue bar represents the timeline; red bar indicates the burst period. “Strength” reflects the intensity of the citation surge relative to the baseline. “Begin” and “End” denote the start and end years of the burst duration. The burst detection was performed using the Kleinberg algorithm with the following parameters: γ = 1.0, minimum burst duration = 2 years, and a time window of 1 year.

Citation burst analysis was used to capture surges in citation activity exceeding base levels within a specific period, offering insight into evolving research trends and influential publications. As shown in Figure 5A, the top 25 references with the strongest citation bursts between 2014 and 2024 reveal two prominent thematic shifts: The early phase (2014–2019) was dominated by studies on opportunistic viral infections and microbiome interactions in IBD. Key contributions included cytomegalovirus (CMV) reactivation and management (strength = 17.3) (Delvincourt et al., 2014), disease-specific alterations of the enteric virome (strength = 31) (Norman et al., 2015), and the therapeutic potential of fecal microbiota transplantation (strength = 12.17) (Moayyedi et al., 2015). The second European Consensus on opportunistic infections remained highly influential throughout this period (strength = 54.79) (Rahier et al., 2014). The recent phase (2020–2024) is overwhelmingly marked by research related to COVID-19 in patients with IBD, particularly focusing on vaccination immunogenicity, clinical outcomes, and medication safety. Noteworthy bursts include studies on attenuated antibody responses under anti-tumor necrosis factor (TNF) therapy (strength = 27.01) (Kennedy et al., 2021), vaccine safety (strength = 15.23) (Polack et al., 2020), and international registry-based outcome studies (strength = 12.71) (Ungaro et al., 2021). This shift underscores how the pandemic has redirected research toward the intersection of COVID-19 and IBD.

### 3.7 Analysis of keywords co-occurrence and cluster

**FIGURE 6:**
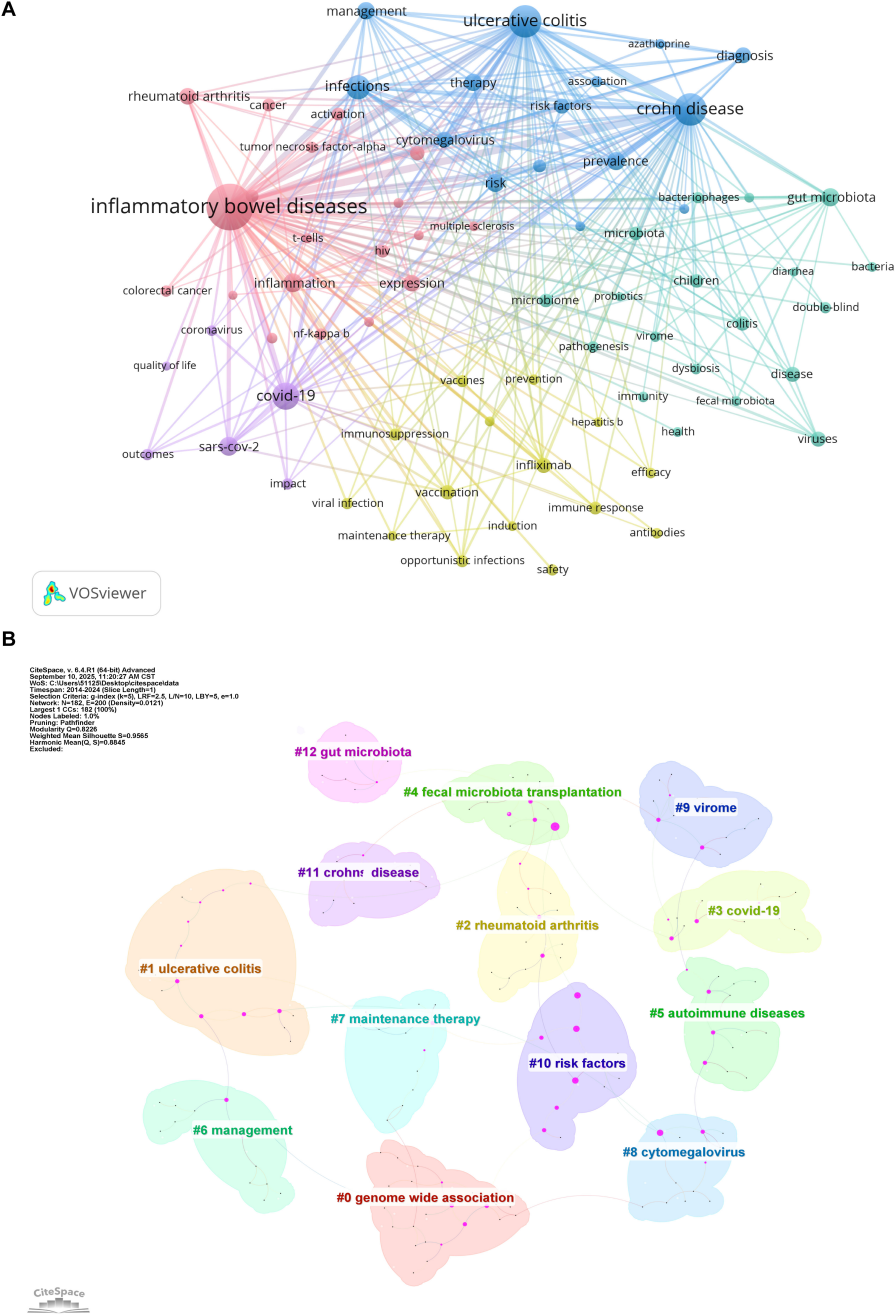
**(A)** Keyword co-occurrence network generated by VOSviewer. Node size indicates keyword frequency; line width represents connection strength. Minimum occurrence threshold = 74. Normalization: association strength. Layout settings: attraction = 2, repulsion = −1. **(B)** Keyword clustering map generated by CiteSpace. Time slice = 1; selection criteria: g-index (*k* = 5), LRF = 2.5, L/N = 10; pruning: pathfinder and sliced networks; *Q* = 0.8226; *S* = 0.9565; labels from LLR.

Keywords represent core research themes, and bibliometric analysis of their co-occurrence helps identify hotspots and trends. This study extracted 11,348 keywords, with the top 20 listed in Table 6. A keyword co-occurrence network was created using VOSviewer (minimum occurrence = 74), identifying 55 core keywords. Five distinct clusters were identified in Figure 6A: (1) The pink cluster focused on immune-inflammatory mechanisms and regulatory signaling pathways, featuring terms like inflammatory bowel diseases, inflammation, tumor necrosis factor-alpha, expression, nf-kappa b, and regulatory T-cells. (2) The yellow cluster addressed epidemiology, risk factors, and clinical management of infections in immunocompromised patients, featuring terms like viral infection, prevention, opportunistic infections, immunosuppression, prevalence, infliximab, vaccine, and hepatitis B. (3) The cyan cluster emphasized microbial communities, dysbiosis in IBD pathogenesis, and microbiome-targeted therapies, featuring terms like gut microbiota, bacteriophages, virome, dysbiosis, probiotics, and pathogenesis. (4) The blue cluster focused on disease-specific risk profiles and clinical management, particularly for viral infections like CMV, featuring terms like ulcerative colitis, Crohn disease, risk factors, cytomegalovirus, infections, management, azathioprine. (5) The purple cluster underscored COVID-19-era challenges in the management of patients with IBD, featuring terms like COVID-19, Severe Acute Respiratory Syndrome Coronavirus 2 (SARS-CoV-2), quality of life, and impact.

**TABLE 6:** The top 20 keywords related to virus-IBD research.

Rank	Keyword	Occurrences	Rank	Keyword	Occurrences
1	Inflammatory bowel diseases	1703	11	Therapy	209
2	Crohn disease	781	12	Management	201
3	Ulcerative colitis	779	13	Rheumatoid arthritis	192
4	COVID-19	521	14	Expression	178
5	Infections	395	15	Diagnosis	166
6	SARS-CoV-2	232	16	Cytomegalovirus	165
7	Inflammation	231	17	Infliximab	152
8	Gut microbiota	227	18	Viruses	148
9	Prevalence	223	19	Disease	143
10	Risk	222	20	Risk factors	142

**FIGURE 7:**
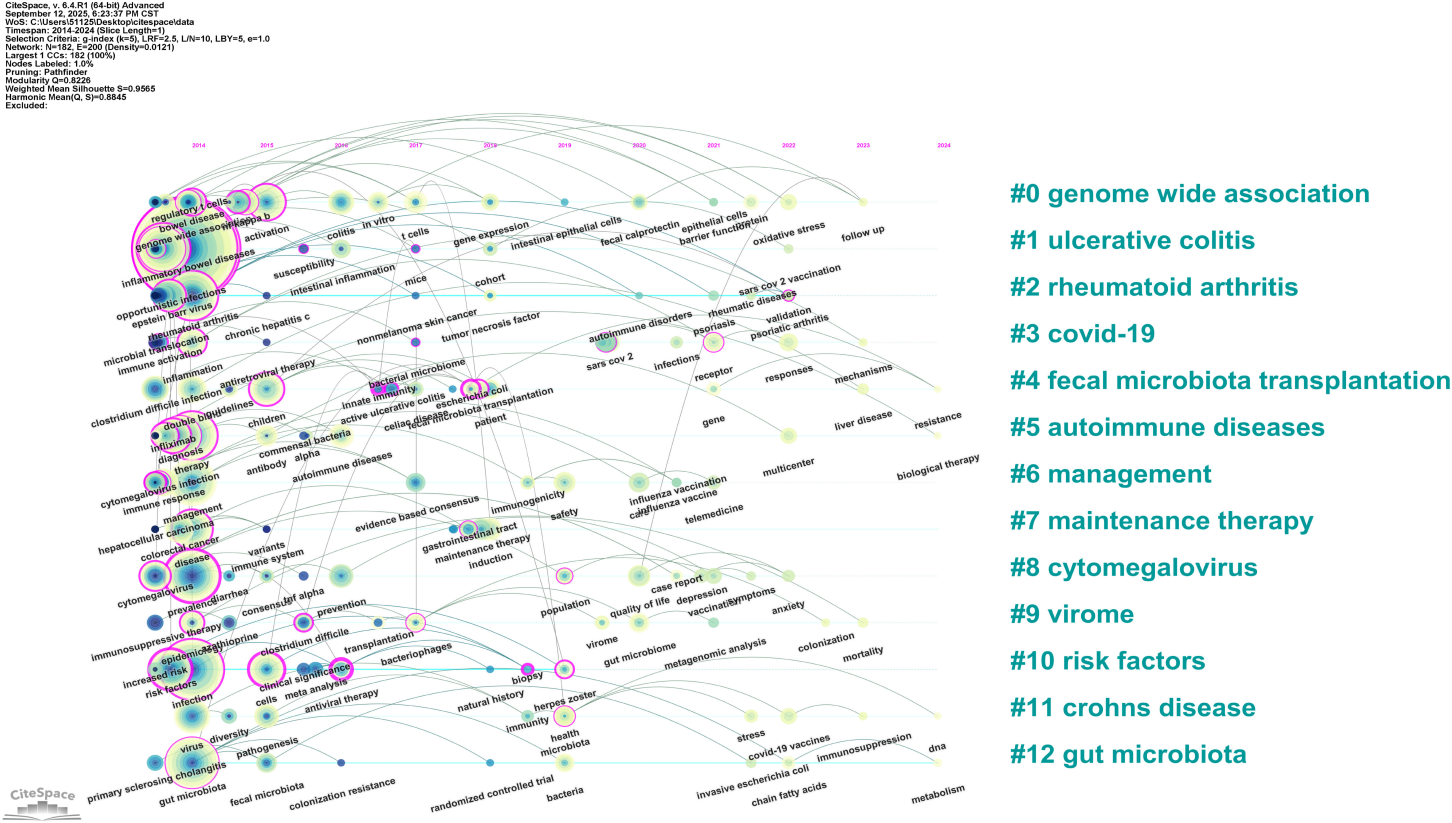
Timeline visualization of keyword clusters. The horizontal axis represents the timeline. Node size reflects keyword frequency, and connecting lines indicate thematic relationships between topics.

CiteSpace was used to generate a keyword cluster timeline to explore temporal evolution, identifying 13 major thematic groups (Figure 6B) genome-wide association, ulcerative colitis, rheumatoid arthritis, COVID-19, fecal microbiota transplantation, autoimmune diseases, management, maintenance therapy, cytomegalovirus, virome, risk factors, Crohn disease, and gut microbiota. The timeline illustrates inter-cluster relationships and chronological distribution, with keywords in the same cluster aligned horizontally (Figure 7). Clusters like risk factors, virome, management, fecal microbiota transplantation, and maintenance therapy were active initially but gradually declined. In contrast, the COVID-19 cluster emerged around 2020, peaked briefly, then reduced in activity. Notably, genome-wide association, cytomegalovirus, and gut microbiota remained active throughout the period, confirming their core status. The fecal microbiota transplantation cluster showed strong links to other groups, indicating interdisciplinary relevance. Recent keywords (2023–2024) point to emerging directions: follow-up studies in genome-wide association, fecal microbiota transplantation resistance, biological therapy for autoimmune diseases, DNA and immunosuppression in Crohn disease, and gut microbiota metabolism.

Keyword burst analysis (Figure 5B) identifies abrupt frequency surges, reflecting research focus shifts. The top 25 bursting keywords (2014–2024) align with reference citation burst trends, dividing into two phases: 2014–2019 (opportunistic viral infections and clinical management) saw core bursts of virus infections (strength = 13.35, 2014–2018), cytomegalovirus infections (7.78, 2014–2017), and immunosuppressive therapy (9.28, 2014–2017)–mirroring citation bursts on CMV reactivation (reference strength = 17.3). Human immunodeficiency virus (HIV) (8.04, 2015–2019) and primary sclerosing cholangitis (8.01, 2017–2019) expanded to viral comorbidities, while meta-analysis (9.3, 2016–2018) and evidence-based consensus (7.69, 2017–2021) aligned with Rahier et al.’s European Consensus (reference strength = 54.79). The 2020–2024 phase pivoted to COVID-19-IBD intersections: COVID-19 (16.86, 2021–2024, strongest burst) and coronavirus (12.11, 2020–2021) echoed COVID-19-focused references; COVID-19 vaccines (8.87, 2022–2024) and vaccination (7.71, 2021–2022) aligned with Kennedy et al. (27.01) and Polack et al. (15.23) on vaccine safety/efficacy; outcome (7.62, 2021–2024) and multicenter (8.03, 2022–2024) mirrored registry studies (Brenner et al. 18.9; Ungaro et al. 12.71); and care (8.85, 2020–2022) and quality of life (7.33, 2020–2022) addressed pandemic-era IBD care continuity.

## 4 Discussion

This bibliometric analysis of 3,225 publications from WoSCC establishes a comprehensive framework to map the research landscape of the virus-IBD field.

### 4.1 General information

The annual publication trends reveal a three-phase evolution: initial fluctuating growth (2014–2019), a sharp surge (2020–2021), and a subsequent decline (2022–2024). These shifts reflect the adoption trajectory of virome research in IBD, influenced by methodological advances in metaviromics, the accelerated integration of high-throughput sequencing, and the transient focus on virology during the COVID-19 pandemic. The recent decline may indicate a post-pandemic refocusing toward mechanistic studies of chronic virus-host interactions and microbiota-virome ecology, alongside persistent challenges in clinical translation. Despite these fluctuations, annual output remained within a stable range, underscoring the maturation of virus-IBD research as a distinct subspecialty.

The USA and China emerged as the dominant contributors, collectively accounting for 46.6% of publications, reflecting their substantial investments and research infrastructure. The USA led in total output (996 publications, 30.88%) and maintained a high average GCS (57.98), indicative of strong scholarly influence. China ranked second in output (507, 15.72%) but had a lower average GCS (27.50), suggesting room for greater international impact. Other high-output countries included Italy, the UK, and Germany. Notably, Canada (avg. GCS 58.29) and Australia (56.98) achieved high per-paper citation rates despite moderate productivity, reflecting focused research excellence. Regional disparities were further evident in LCS. Belgium, France, and Canada led in average LCS, whereas several East Asian nations and emerging economies (e.g., China, South Korea, Iran) showed lower regional citation visibility. Collaboration networks were most intensive among North American and Western European countries, highlighting the role of established research ecosystems in fostering high-impact partnerships.

Institutional analysis underlined the dominance of the institutions from the USA, which constituted 13 of the top 20 producers. The Mayo Clinic led in publication volume (73), the Icahn School of Medicine at Mount Sinai had the highest total LCS (723), and UCSF achieved the highest total and average GCS (9,624 and 240.60, respectively). Institutions such as Humanitas University (Italy) also demonstrated notable regional impact, with an exceptional average LCS (16.76). Network visualization confirmed that leading institutions–including the Mayo Clinic, Icahn School of Medicine, and Harvard Medical School–serve as central hubs within collaborative frameworks, bridging basic and clinical research domains.

Author analysis identified key contributors: Francis A. Farraye, Silvio Danese, and Jean-Frederic Colombel were among the most prolific, while Shaji Sebastian and Siew C. Ng led in LCS and GCS, respectively. Jean-Frederic Colombel had the highest H-index (135), reflecting sustained academic influence. The co-authorship network revealed four major clusters, with tightly integrated collaborations around Jean-Frederic Colombel, Silvio Danese, and Francis A. Farraye, while the cluster led by Suk-Kyun Yang (South Korea) was relatively isolated, suggesting regionally concentrated activity.

Journals such as *Inflammatory Bowel Diseases* (135 articles), *Frontiers in Immunology* (100), and *Journal of Crohn’s* & *Colitis* (81) were the most prolific. *Gastroenterology* had the highest average GCS (244.64) and, with *Gut*, the highest Impact Factors, affirming the role of leading clinical journals in high-impact virus-IBD research. *Frontiers in Immunology* ranked second in total GCS (3,931), highlighting its niche focus on immunovirological mechanisms. Journals including *International Journal of Molecular Sciences* and *Scientific Reports* exhibited strong recent impact based on H5 index, illustrating the multidisciplinary nature of the field.

### 4.2 Hotspots and Frontiers

Keyword co-occurrence and reference analysis reveal the conceptual evolution and emerging priorities within virus-IBD research. Two major thematic shifts were identified: an initial focus on opportunistic infections, virome ecology, and clinical management (2014–2019), followed by a pronounced pivot toward COVID-19-related outcomes, vaccination, and pandemic-era care (2020–2024). Concurrently, keyword clustering highlights persistent interest in immune mechanisms, viral pathogenesis, and microbiome interactions. These dynamics frame three current research hotspots: gut virome mechanisms and therapy, clinical strategies against opportunistic viral infections and IBD management during COVID-19-each supported by robust citation activity and keyword emergence, as further discussed below.

#### 4.2.1 Mechanisms and therapeutic strategies of gut virome regulation in IBD pathogenesis

The gut virome plays a critical role in the pathogenesis of IBD through bacteriophage-mediated modulation of bacterial communities and immune interactions (Liang et al., 2021; Tun et al., 2024). Metagenomic analyses show consistent structural changes in IBD, including reduced viral α-diversity, increased *Caudovirales* bacteriophages, and disease-specific markers such as mucosal *Hepadnaviridae* in UC and *Hepeviridae* in CD (Wagner et al., 2013; Clooney et al., 2019; Ungaro et al., 2019; Zuo et al., 2019; Liang et al., 2020). Active CD is further characterized by a reduced ratio of lytic to temperate phages (Cao et al., 2024). These virome perturbations correlate directly with dysbiosis severity and show diagnostic potential (Gulyaeva et al., 2022; Tian et al., 2024), indicating their active role in pathogenesis (Norman et al., 2015; Shkoporov et al., 2019).

The interaction between the virome and the immune system occurs through distinct pathways. Pro-inflammatory processes are characterized by phage-mediated release of pathogen-associated molecular patterns (PAMPs), which activate Toll-like receptor 9 (TLR9)-dependent secretion of interferon gamma (IFN-γ), thereby intensifying mucosal inflammation (Gogokhia et al., 2019; Buttimer et al., 2023). Additionally, host genetic factors, such as mutations in ATG16L1 associated with CD, can interact with viral infections like norovirus to exacerbate pathological conditions (Nakase et al., 2021; Hamade et al., 2024; Zhang et al., 2024). Conversely, protective mechanisms mediated by enteric eukaryotic viruses enhance intestinal barrier integrity through the suppression of nuclear factor kappa B (NF-κB) via TLR3 and TLR7 pathways, particularly in UC (Yang et al., 2016; Nishio et al., 2021; Adiliaghdam et al., 2022).

These insights inform emerging therapeutic strategies. Phage-based precision therapy can target and eliminate specific pathogens (e.g., *Streptococcus gallolyticus*), reducing intestinal inflammation through selective bacterial lysis (Li et al., 2024; Zhao et al., 2025). Fecal virome transplantation has shown efficacy in resolving refractory infections in patients with IBD (Rasmussen et al., 2024). However, translational challenges related to delivery efficiency and safety require validation through large-scale clinical trials.

#### 4.2.2 Clinical challenges and management strategies of opportunistic viral infections

Bibliometric analysis identifies opportunistic viral infections-particularly Epstein-Barr virus (EBV), CMV, Hepatitis B virus (HBV)/Hepatitis C virus (HCV), and HIV–as a major research cluster. Patients with IBD are at elevated risk due to immunosuppressive therapies (e.g., corticosteroids, thiopurines), malnutrition, and comorbidities, underscoring the importance of guideline-driven management (Dave et al., 2014; Rahier et al., 2014).

EBV seroprevalence in patients with IBD reaches 79.4%–100%, and CMV prevalence is 34.5%, markedly higher than those in healthy populations (Linton et al., 2013; Wang et al., 2022). Thiopurine use and chronic inflammation increase the risk of EBV- or CMV-related complications, including hemophagocytic lymphohistiocytosis and lymphoproliferative disorders (Deneau et al., 2010; Wu et al., 2019). EBV co-infection may mimic or exacerbate CD (Zhang et al., 2022a), while CMV is frequently detected in steroid-refractory UC and contributes to glucocorticoid resistance and histopathological severity (Siegmund, 2017; Kwon et al., 2022).

Immunosuppression also elevates HBV/HCV reactivation risk, particularly under anti-TNF therapy, necessitating preemptive screening, vaccination, and antiviral prophylaxis when indicated (Singh et al., 2022). Double-dose HBV vaccination improves seroconversion in these patients (Gisbert et al., 2012). HIV-IBD interplay presents a paradox: HIV-induced CD4^+^ depletion may attenuate intestinal inflammation in some cases (“immune exhaustion remission”), though meta-evidence remains conflicting (Guillo et al., 2022; Huang et al., 2024). Nevertheless, opportunistic infections remain more common in HIV-positive patients with IBD (Calafat et al., 2025).

Preventive strategies emphasize systematic screening (HBV, HCV, HIV, Varicella-Zoster virus) before immunosuppression, alongside inactivated vaccination where applicable (Farraye et al., 2017; Lamb et al., 2019; Caldera et al., 2025). Live vaccines are contraindicated during active immunosuppression (Kucharzik et al., 2021). During active viral infection, immunosuppressants should be withheld and antiviral therapy initiated. While antivirals are effective in CMV colitis, their role in EBV-related disease is less well-defined, highlighting a need for randomized trials (Ciccocioppo et al., 2015).

#### 4.2.3 Management of IBD during the COVID-19 pandemic

Keyword burst analysis revealed that pre-COVID-19 research focused on virome profiling, opportunistic infections, and virus-mediated immune-inflammatory pathways. The pandemic significantly reshaped research priorities, consistent with earlier bibliometric studies (Veisman et al., 2022; Wang et al., 2023). Research emphasis shifted toward COVID-19 susceptibility and outcomes in patients with IBD, as well as vaccination challenges during immunotherapy. Post-pandemic, attention has increasingly turned to the interaction networks between the gut microbiome, virome, and IBD pathogenesis.

Large-scale epidemiological studies indicate that patients with IBD have infection rates comparable to the general population (Monteleone and Ardizzone, 2020). However, the risk of severe COVID-19 is heightened in those with active disease, advanced age, comorbidities, or corticosteroid treatment (Brenner et al., 2020; Ungaro et al., 2021; Ricciuto et al., 2022). Immunotherapy management and vaccination optimization became critical priorities, leading to guidelines from the British Society of Gastroenterology and the American College of Gastroenterology (Kennedy et al., 2020; Rubin et al., 2020), later refined through international consensus on evidence-based vaccination strategies (Lin et al., 2022). Cohort studies confirm vaccine safety and efficacy without IBD exacerbation (Lev-Tzion et al., 2022; Summa and Hanauer, 2023; Viazis et al., 2023). This evidence base provides a framework for managing future infectious disease outbreaks in IBD, guiding immunotherapy and vaccine response optimization.

### 4.3 Limitations

This study offers a systematic bibliometric perspective on virus-IBD interactions but has several limitations. First, only English-language publications were included, which may overlook geographically distinctive virome patterns reported in non-English literature. This introduces potential bias in regional representation and keyword analysis, overrepresenting outputs from English-speaking regions. Second, the analysis relied solely on WoSCC. Although WoSCC was chosen for its well-curated metadata and compatibility with standard bibliometric tools, it underrepresents certain regions and journals compared to broader databases such as Scopus or PubMed, which may affect the geographic and thematic comprehensiveness of the results. Third, keyword co-occurrence analysis is constrained by author-defined terminology, which often includes inconsistent nomenclature leading to fragmented conceptual clusters. Despite applied normalization procedures, infrequent terms remain susceptible to semantic fragmentation. Furthermore, co-occurrence networks simplify complex semantic relationships, and algorithmic pruning may emphasize specific citation patterns. Finally, the inclusion of a considerable number of review articles may inflate citation counts, as they are typically cited more frequently than original articles. This could confound the identification of seminal original research. To address these issues, future studies should: (1) incorporate multiple databases to improve geographic and thematic coverage; (2) employ natural language processing for more consistent term standardization; and (3) test robustness using varied analytical parameters and document-type stratification. Nonetheless, multidimensional validation supports the stability of the core conclusions presented here.

## 5 Conclusion

This bibliometric analysis systematically delineates the knowledge structure and evolving trends in virus-IBD research, identifying three predominant research areas. The findings reflect an ongoing evolution from pathogen-specific studies toward a more integrative understanding of virus-host-microbiota interactions. The results provide actionable insights for both clinical practice and research strategy: they highlight promising translational domains such as virome-based biomarkers and phage therapy, while also revealing underexplored topics-such as cross-regional virome disparities-that warrant targeted funding. Future research should prioritize: (1) mechanistic investigations using multi-omics approaches to identify key regulatory nodes through which viruses influence gut immune homeostasis, with emphasis on innate immune receptor-virome interactions; (2) developing innovative virome-targeted therapies, including phage-based interventions; (3) advancing clinical translation by integrating virome-derived biomarkers into personalized therapeutic frameworks, such as virome-guided subtyping of IBD. This evidence-based framework helps identify interdisciplinary research gaps, supports resource allocation, and informs public health policy.

## Data Availability

The raw data supporting the conclusions of this article will be made available by the authors, without undue reservation.
